# Engineering techniques to dendrite free Zinc-based rechargeable batteries

**DOI:** 10.3389/fchem.2022.1018461

**Published:** 2022-09-29

**Authors:** Ababay Ketema Worku

**Affiliations:** Bahir Dar Energy Center, Bahir Dar Institute of Technology, Bahir Dar University, Bahir Dar, Ethiopia

**Keywords:** rechargeable battery, Zn dendrites, Zinc-based batteries, dendritic morphology, Zinc anode, energy storage and conversion

## Abstract

Rechargeable Zn-based batteries (RZBs) have garnered a great interest and are thought to be among the most promising options for next-generation energy storage technologies due to their low price, high levels of safety, adequate energy density and environmental friendliness. However, dendrite formation during stripping/plating prevents rechargeable zinc-based batteries from being used in real-world applications. Dendrite formation is still a concern, despite the fact that inhibitory strategies have been put up recently to eliminate the harmful effects of zinc dendrites. Thus, in order to direct the strategies for inhibiting zinc dendrite growth, it is vital to understand the formation mechanism of zinc dendrites. Hence, for the practical application of zinc-based batteries, is essential to use techniques that effectively prevent the creation and growth of zinc dendrites. The development and growth principles of zinc dendrites are first made clear in this review. The recent advances of solutions to the zinc dendrite problem are then discussed, including strategies to prevent dendrite growth and subsequent creation as much as possible, reduce the negative impacts of dendrites, and create dendrite-free deposition processes. Finally, the challenges and perspective for the development of zinc-based batteries are discussed.

## 1 Introduction

Numerous significant advancements have been made in the development of electrochemical energy storage systems over the past several years (Goh et al., 2013). Batteries are an example of an electrochemical energy storage technology that can store electric energy as chemical energy and convert the chemical energy to electric energy as needed (Worku et al, 2021a; Khamsanga et al., 2019). Thus, battery technologies can increase the use of renewable energy sources while reducing the use of limited fossil fuels (Li et al., 2016). Battery technology has so far been applied to stationary energy storage and power batteries. As an illustration, lithium-based batteries are typically used as the power source for electric vehicles, mobile phones, laptops, and other mobile devices (Worku et al., 2022). The use of lithium-ion batteries to store renewable energy is currently receiving a lot of interest despite the fact that these batteries pose safety risks, are expensive, and have a low energy density. As a result, numerous high-safety battery types have been suggested and thoroughly studied. Due to their high levels of safety and affordability, green and sustainable energy storage systems made of aluminum, zinc, potassium and sodium have recently gained a lot of attention (Zhong et al., 2021). Due to their plentiful resources, environmental friendliness, and high energy density, zinc-based rechargeable batteries, such as, Zn-Ni batteries, Zn-MnO_2_ batteries (Sumboja et al., 2015), zinc-ion batteries and zinc-based flow zinc-air batteries, are thought to be the most promising energy storage devices to replace lithium batteries (HabtuGabbiye et al., 2022).

Additionally, zinc has high capacity (5,854 Ah·L^−1^ and 820 Ah·kg^−1^), good electrochemical reversibility [0.762 V relative to the standard hydrogen electrode (SHE)], and does not readily corrode even in alkaline conditions (Abbasi et al., 2019). Zinc has a number of other advantages when used as an electrode material, including a high specific energy, a high power density, a low redox potential, nontoxicity, recyclability, and low cost. Zinc is currently one of the most widely utilized electrode materials because zinc-based batteries often have high energy density, low cost, high discharge voltage, and good environmental benignity. Zinc-based batteries are promise for the next wave of energy storage technologies because of these characteristics (Worku et al., 2021b). Notably, zinc oxides and their combinations can also function in addition to pure zinc. However, the issues of dendritic growth, self-corrosion, and morphological change are still unresolved, which has a significant impact on the efficiency of zinc plating and striping and the service life of zinc electrode (Worku et al., 2021c).

The ability to suppress dendrite growth in particular is crucial for improving the Coulombic efficiency (CE) and stability of zinc-based batteries (Hosseini et al., 2018). Due to unequal zinc deposition during the charging process of secondary zinc-based batteries, zinc dendrites are formed. The performance and lifespan of zinc-based batteries are significantly impacted by the presence of zinc dendrites (Fu et al., 2016).

**FIGURE 1:**
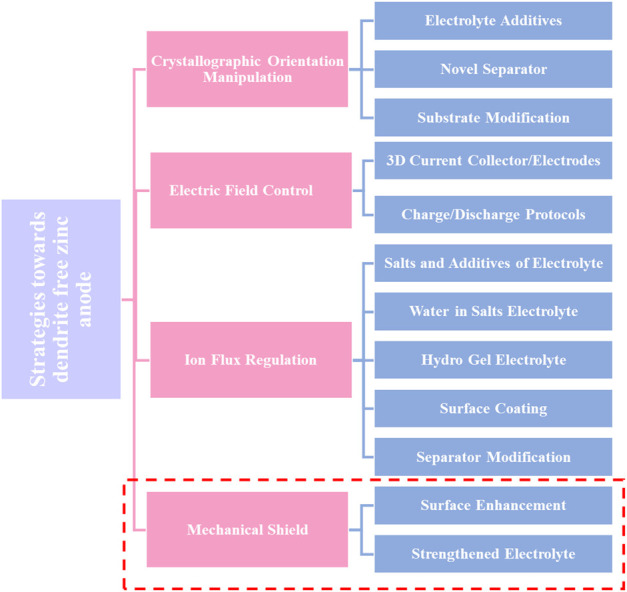
Schematic representation of the materials used in RZIBs to stabilize Zn anodes. Reproduced under the terms of the CC-BY Creative Commons Attribution 4.0 International license (Q. Li et al., 2020). Copyright (2022), Energy Materials.

In addition, zinc dendrites readily detach from electrodes in alkaline media, resulting in a decline in battery capacity and efficiency. Additionally, when zinc dendrites continue to grow, they eventually come into direct contact with the anode and cathode, creating a short circuit and the eventual collapse of the battery (Lee et al., 2016). Additionally, the dendritic shape might increase the zinc electrode’s specific surface area, which promotes zinc corrosion and lowers the zinc consumption rate. In addition, zinc dendrites would easily separate from the electrode surface to generate “dead” zinc as a result of the weak adhesion, reducing the battery’s capacity. In recent years, numerous strategies have been proposed to reduce uneven zinc deposition and enhance the cycling performance of zinc-based batteries (Ayele et al., 2021b). Rechargeable zinc-air batteries are still far from becoming commercially available for a number of reasons, one of which is the fact that the zinc anode has a poor CE as a result of the development of zinc dendrites (Zuo et al., 2021). Moreover, for a very long time, Li metal batteries have been plagued by dendrite formation and the dead Li that results from it. Recent research has suggested a novel redox mediator-based approach to lithium restoration. However, using some redox mediators frequently results in the unwanted side effect of significant self-discharge. Chen et al. (2022), reported a selection principle of redox mediators for reactivating dead Li in lithium metal batteries. This approach may both successfully reactivate the dead Li and decrease self-discharge. These strategies include adding additives to the electrolyte or anode metal, optimizing operating parameters, and removing the detrimental effects of dendrite growth. The main concepts behind these techniques can be broken down into three groups (I) approaches to prevent zinc dendrites from forming and growing further as much as possible; (II) approaches to reduce the negative effects caused by zinc dendrites; and (III) approaches to get rid of zinc dendrites and ultimately create a deposition process without them (Chu et al., 2022). The first approach, while currently the most popular way to address the dendritic problem, also has significant downsides, like greater electrode polarization. Although the second and third approaches are more difficult to implement, their effectiveness is thought to be sufficient, and certain concepts and real-world applications have been suggested (K. Wang Anran et al, 2020a). Despite the fact that these techniques have made significant strides in slowing zinc dendrite formation, it is still unknown what causes homogeneous zinc deposition. In order to provide workable inhibitory strategies for morphological control of electrodeposited zinc, additional research and knowledge of the process underlying zinc dendrite formation are therefore required (Worku et al., 2021d). In order to stabilize the Zn anodes, various techniques have been used. These include surface modification, structural design, and electrolyte control (Figure 1). These methods serve to improve the electrochemical performance of RZIBs by successfully suppressing Zn dendrite development and/or side reactions. This review outlines the theories and approaches for addressing zinc dendrite problems and reducing their negative effects (Lu et al., 2018). As a result, it can serve as a thorough reference to guide the advancement and practical use of zinc-based batteries in the future.

## 2 Overview of Zn anode

Despite the development of alternate substitutes, Zn anodes remain the most optimal anodes for RZBs due to their incomparable benefits. The issues with Zn anodes may be found in numerous laboratory investigations that often include high anode sources, low current densities, and restricted loading mass in the cathode (Wang et al., 2015). Due to its “hostless” nature and uniform stripping and plating, Zn anodes inevitably experience dendritic difficulties, much like many other metal anodes. Metal anodes based on stripping and plating mechanism witnessed unlimited volume change as opposed to standard graphite anodes based on insertion mechanism since this “hostless” nature can lead to uncontrollable dendrites growth. In addition, there are other problems with the Zn anode aqueous system, such as corrosion, passivation, and hydrogen evolution, which are worse in alkaline electrolytes. Typically, Zn metal is used directly as the anode of RZIBs. Due to their security and affordability, RZIBs with Zn metal anodes have a lot of potential for large-scale energy storage. The anode-electrolyte interface problems are the main reason why their practical performances are still below expectations. The following sections discuss these problems, which mostly concern dendritic formation and side reactions on the surface of zinc anodes (Li et al. 2020a).

## 3 Dendrite formation and side reactions

### 3.1 Formation of dendrite

The reaction mechanism of a Zn anode in the mild aqueous electrolyte can be summed up as follows (Wang et al., 2021):

Zn^2^ + (aq) + 2e^-^ ↔ Zn(s) (1)

**FIGURE 2:**
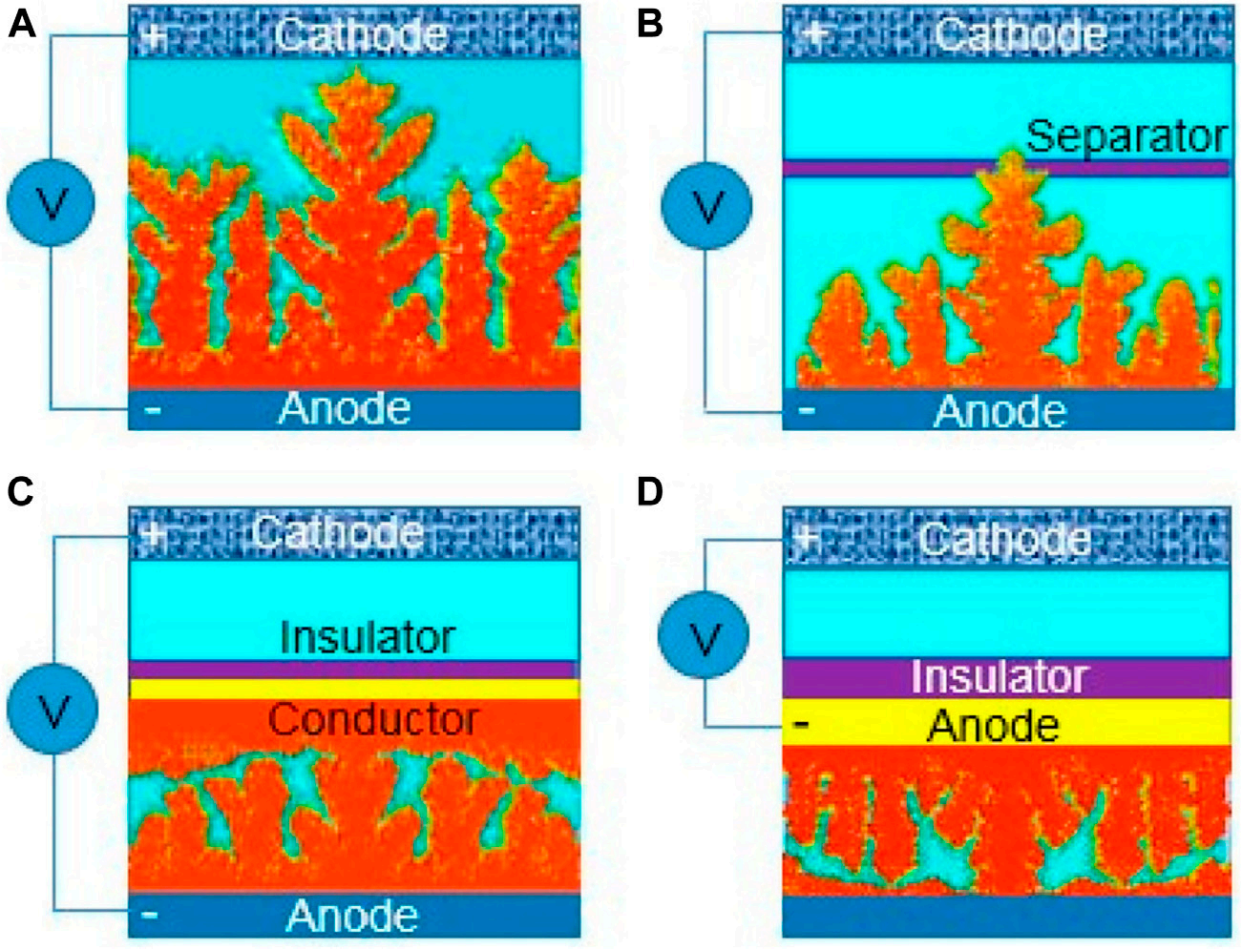
Dendrite growth of depositing zinc. **(A)** Dendrite growth causing short circuit of the batteries, **(B)** dendrite growth puncturing a separator, **(C)** partially conductive separator guiding dendrite growth, and **(D)** insulator encapsulating anode-reversing dendrite growth. Reproduced under the terms of the CC-BY Creative Commons Attribution 4.0 International license (Zhang et al., 2022). Copyright (2022), Energy Materials.

**FIGURE 3:**
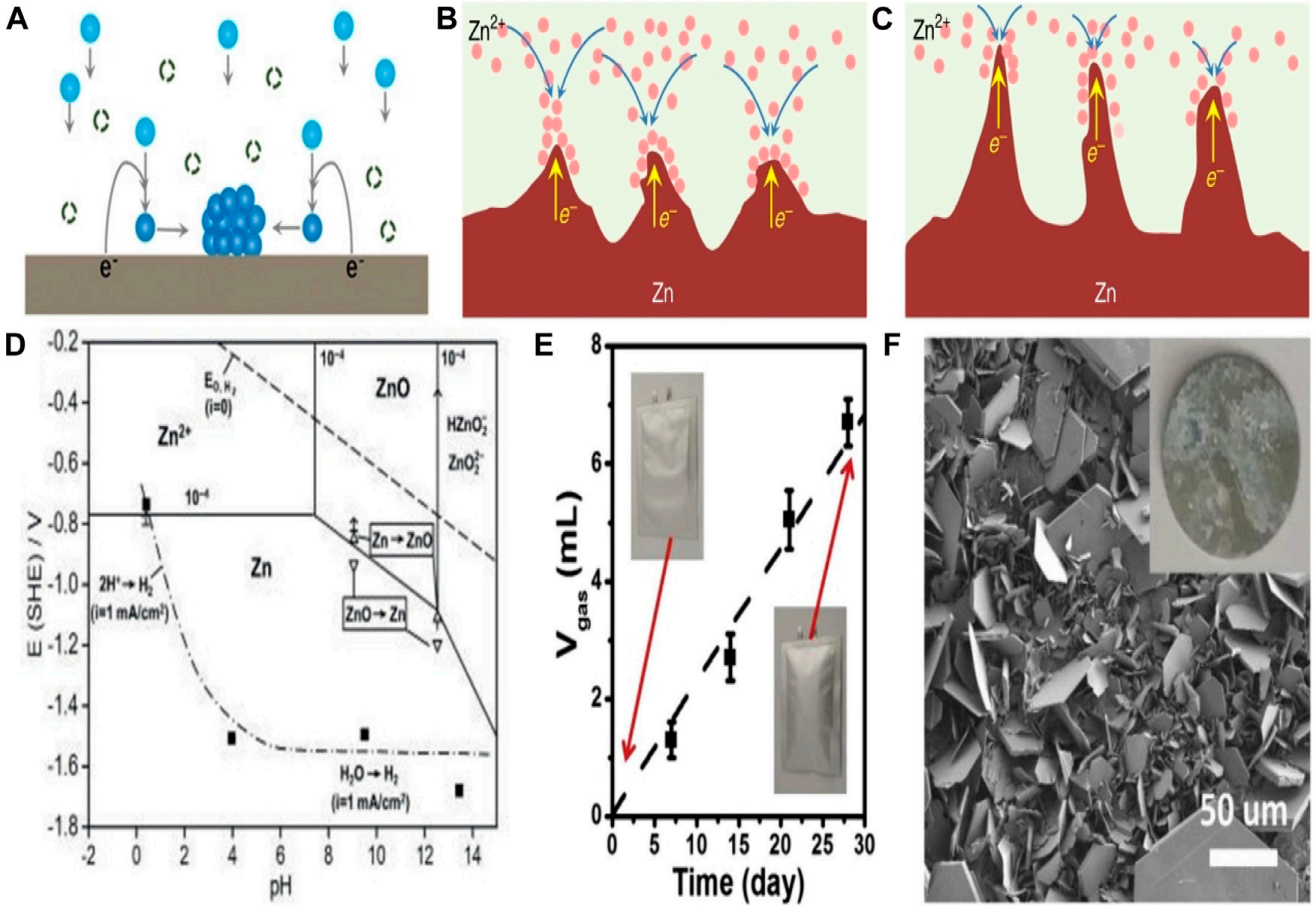
Schematic representation of the creation of Zn atomic clusters during unconstrained 2D Zn^2+^ diffusion. **(A)** Schematic representation of the tip effect in **(B)**. Dendrite formation is option **(C)**. Pourbaix diagram of the 10^–4^ M Zn^2+^ system in Zn/H_2_O. **(D)** The gas evolution of a Zn symmetric cell in a 3 M ZnSO_4_ electrolyte at various resting times. **(E)** The Zn anode’s surface morphology after 30 days of immersion in 3 M ZnSO_4_ electrolyte. **(F)** Reproduced under the terms of the CC-BY Creative Commons Attribution 4.0 International license (Zhang et al., 2022). Copyright (2022), Energy Materials.

Diffusion, adsorption, growth and nucleation are the usual four phases that Zn^2+^ goes through during electrodeposition. The Zn anode surface microenvironment can affect these activities. Particularly, Zn anodes’ surfaces are not atomically smooth, which may lead to irregular electric field distribution, heterogeneous ion flux distribution, and various nucleation barrier sites (Guo et al., 2020). Therefore, under the unconstrained 2D Zn^2+^ diffusion, Zn^2+^ is more likely to adsorb and accumulate on the higher active sites (Figure 2A). Zn atomic clusters would then develop as a result of the Zn^2+^ nucleating on these locations. The distribution of the produced Zn atomic clusters on the surface of Zn is heterogeneous, which exacerbates the unequal field distribution (Zeng et al., 2019). Due to the tip effect, these clusters can also act as small protrusions with greater curvature and stimulate Zn dendrite formation (Figures 2B–D). A number of risks would be brought by the expanding Zn dendrites (Zuo et al., 2021). Due to the Zn dendrites’ porous and flimsy 3D shape, fresher Zn could come into contact with aqueous electrolytes, increasing the potential for side reactions (Xie et al., 2020). Additionally, because of the poor connection between the dendrites and anodes, the dendrites are vulnerable to rupturing away from the Zn substrate and turning into “dead Zn.” The insulating byproduct layer and inactive “dead Zn” increase the battery’s internal resistance and polarization (Figures 3A–C). Along with “dead Zn,” certain dendrites may develop continuously until they pierce the separator, which will result in a short circuit (Li et al. 2020b).

### 3.2 Rechargeable Zn-based batteries side reactions

Other negative issues with Zn anodes include side reactions, such as passivation, corrosion, and HER, in addition to dendritic formation (Liu et al., 2019b). One of these is the Zn anode’s thermodynamic instability in an aqueous solution, which is the main contributor to the development of hydrogen (Li et al., 2022). The reaction of hydrogen evolution can be demonstrated as follows:

2H_2_O + 2e^-^ → 2OH^-^ + H_2_↑ (2)

Surface corrosion from hydrogen evolution can result from chemical or electrochemical reactions. Normally, electrochemical cells are destroyed by hydrogen evolution because it raises internal pressure and causes the sealing to fail (Han et al., 2020). The Pourbaix diagram (Figure 3D) shows that in the whole pH range, Zn^2+^/Zn has a lower equilibrium potential than H_2_O/H_2_ does. Because of the thermodynamic activity of Zn in an aqueous solution, HER tends to develop on the surface of Zn metal anodes *via* chemical or electrochemical processes (Figure 3E **)**. As a result, when RZIBs are charged, HER competes with the Zn plating and there would be an associated Zn corrosion process. HER raises the battery’s internal pressure, which may further increase polarization, cause the battery to swell and even rupture. Due to the accumulating OH^−^, HER also causes a rise in pH at the anode surface. In order to generate byproducts with limited solubility, such as Zn(OH)_2_, Zn_4_SO_4_(OH)_6_xH_2_O (ZHS), etc., the continuously rising OH^−^ would further react with Zn^2+^ and the anion of Zn salts (Figure 3F). These byproducts, which act as electrical insulators and passivate the Zn surface to prevent additional Zn plating or peeling, block the sites. In an alkaline environment, the Zn anode’s passivation usually occurs by generating insulating ZnO on the anode’s surface, which prevents the anode from engaging in further electrochemical action. They can’t stop the additional HER and Zn corrosion on the Zn anodes because they are currently free in the framework (Tian et al., 2021). Zn and electrolytes around anodes are thus continuously consumed, which results in a lower CE. Additionally, the corrosion- and passivation-induced rough and uneven surface may hasten the formation of Zn dendrites (Mo et al., 2022).

### 3.3 Reducing side reactions

**FIGURE 4:**
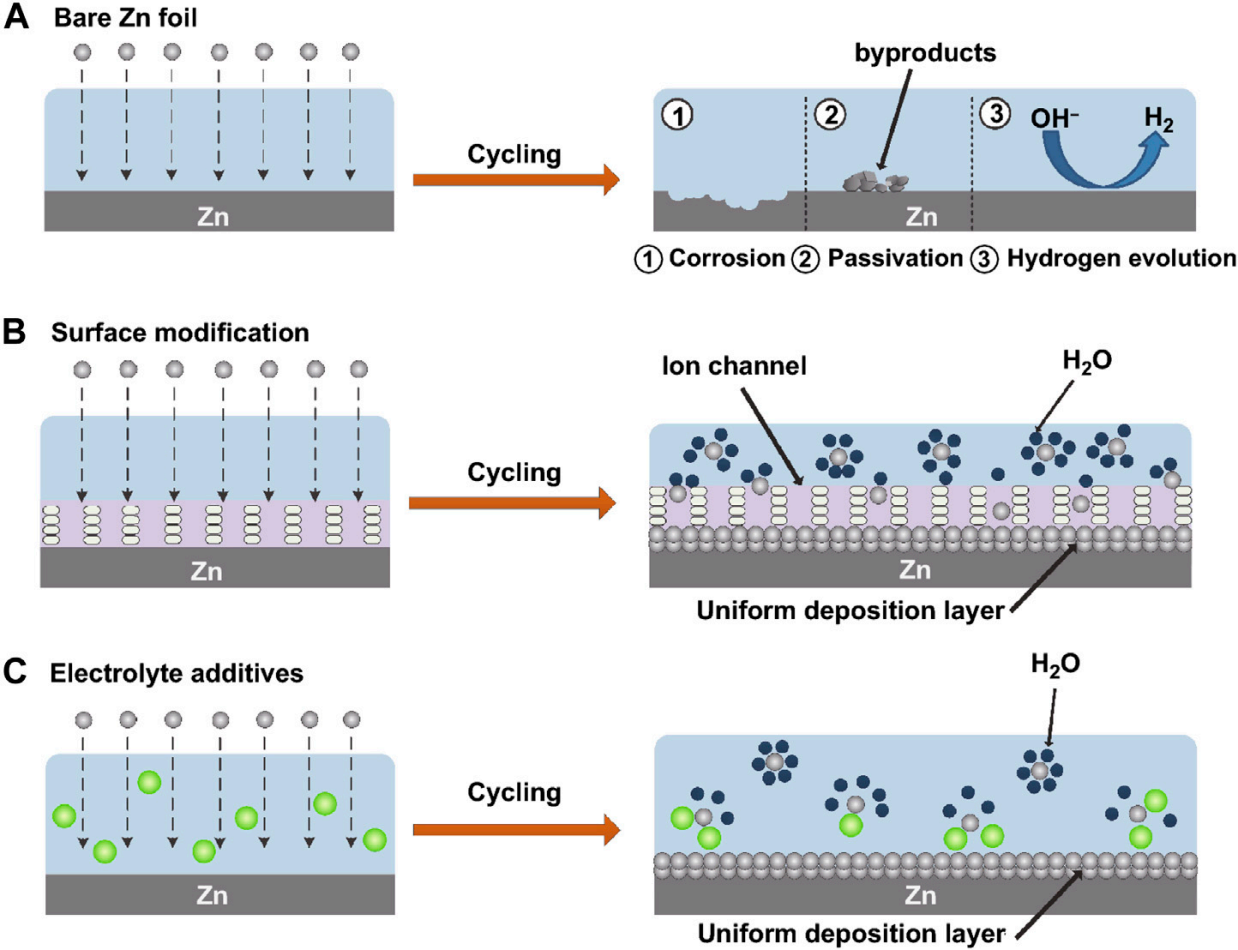
The passivation, HER and corrosion, on bare Zn. **(A)** A schematic illustration of coated Zn’s morphological development. **(B)** Schematic representation of the Zn ion deposition morphology after electrolyte additions. **(C)** CC-BY Creative Commons Attribution 4.0 International license was used to permit this reproduction (Li et al., 2022). Copyright (2022), Springer Nature.

The Zn anode’s surface area grows when Zn dendrites develop. Surface-dependent processes that consume active Zn continuously and significantly lower battery capacity include corrosion reactions and the hydrogen evolution reaction. The gas will result in volume expansion of the batteries when the side reaction of hydrogen evolution takes place in a nearby high-energy location. In addition, insoluble Zn(OH)_2_ is created and attaches to the metal Zn surface, inducing surface passivation of the fresh Zn, as the local OH^−^ concentration rises. This leads to a poor plating/stripping CE by decreasing the anode’s conductivity, raising the interface impedance, and decreasing the active Zn nucleation sites. The performance and longevity of batteries are put at risk by these irreversible hydrogen evolution, corrosion, and passivation side processes, which fundamentally deplete limited electrolyte and Zn ions (Wang et al, 2020a). A schematic representation representing Zn corrosion, passivation, and hydrogen development can be seen in Figure 4A.

#### 3.3.1 Reducing active water

In aqueous electrolytes, Zn^2+^ and six water molecules combine to generate hydrated Zn^2+^([Zn(H_2_O)_6_]^2+^), which is the principal cause of side reactions. Before being reduced on the surface of the Zn anode, [Zn(H_2_O)_6_]^2+^ must go through a desolvation process, which inexorably results in direct contact between the Zn anode and water molecules and sets off side reactions. In order to increase the hydrogen evolution potential of metal Zn and lessen the corrosion response, it was discovered that adding atomic groups or solid electrolyte interface layers to the anode surface is advantageous. Additionally, the interfacial layer either directly blocks contact between the electrolyte and the Zn anode or lowers the amount of water molecules that are allowed to desolvate onto the Zn surface (Li et al., 2013). Following the protective layer, the Zn deposition is extremely uniform, as illustrated in Figure 4B.

#### 3.3.2 Modulating coordination status

The parasitic water reduction during Zn deposition is sped up by the high overpotential created by the robust Coulomb contacts between the solvated Zn^2+^ and its surrounding H_2_O shell. As a result, a passivation layer and the evolution of H are encouraged. The strength of the connection between the Zn^2+^ ions and solvated H_2_O needs to be reduced in order to prevent water reduction and Zn dendrites. A quick and convenient method for improving the electrolyte composition is to add particular chemicals (Figure 4C). Some additions can solvate with Zn ions preferentially, substitute H_2_O in the Zn^2+^ solvated sheath, or remove H_2_O entirely (Liu et al., 2019b).

## 4 Engineering approaches of Zn metal anodes surface modification

**FIGURE 5:**
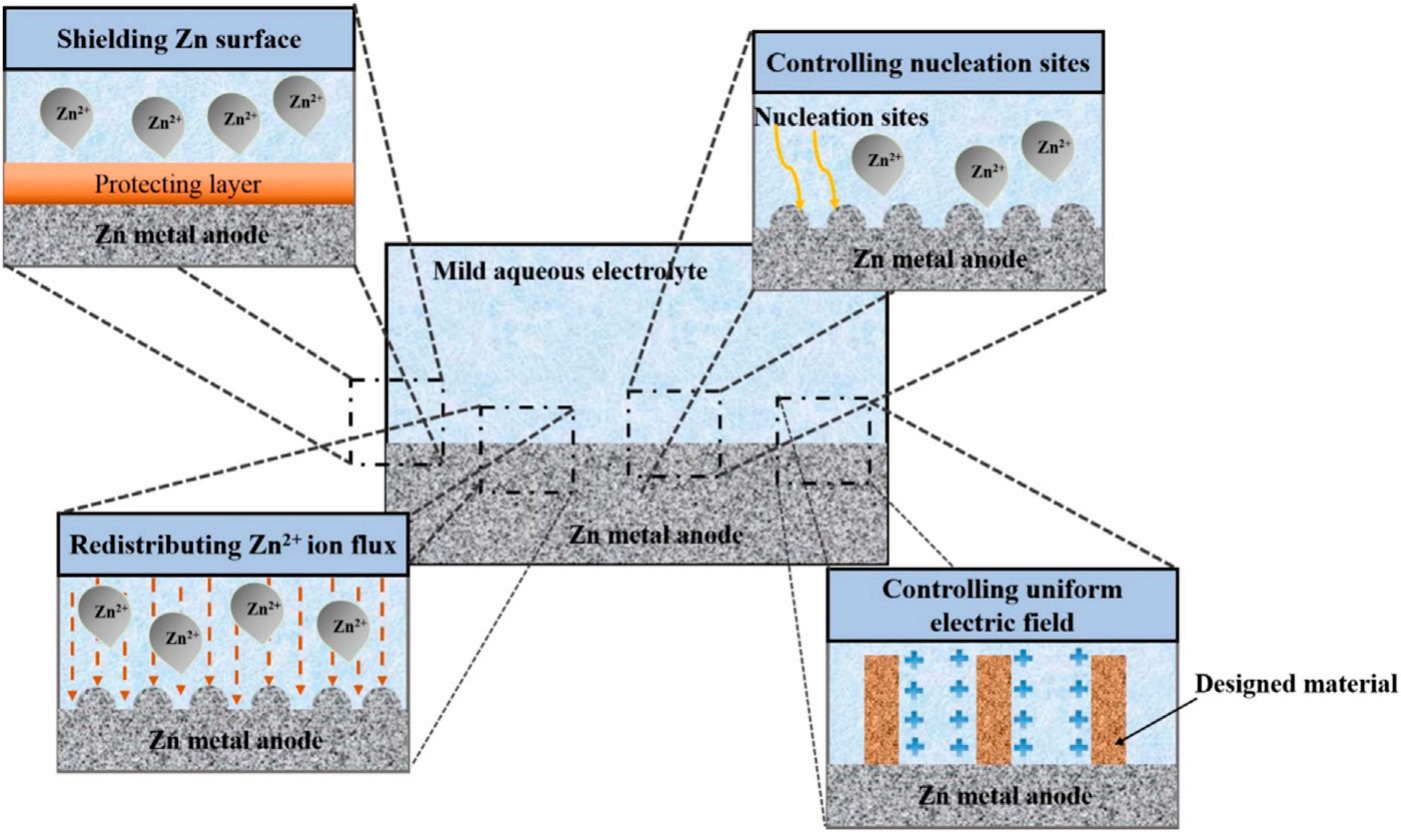
Techniques for modifying Zn metal anodes to improve their electrochemical performance. Reproduced with permission (Huy, Hieu, and Hur 2021). Copyright (2021), MDPI.

The Zn metal electrode surface’s structure has a significant impact on the electrochemical performance of RZIBs. The surface of Zn metal electrodes has therefore been modified using a variety of techniques. The dominant crystallographic orientation and initial anode surface texture will have a significant impact on the following electrochemical behavior. The mass transfer process, which is primarily driven by the electric field and concertation gradient throughout the battery cycling process, commands the dendrite’s creation. From a mechanical standpoint, the strength of the interaction with physical shielding will have an impact on the dendrite growth. These techniques can be divided into four basic categories: Mechanical shielding, ion flow regulation, electric field control, and manipulation of crystallographic orientation (Tian et al., 2021). These techniques can be divided into several key categories, such as shielding the Zn metal to avoid side reactions, controlling the Zn deposition behavior, and producing a consistent electric field as shown in Figure 5. For future reference, the unexplored mechanical viewpoint that mechanical shielding inhibits dendrite formation is listed below. This section discusses a variety of techniques within each area.

### 4.1 Electric field control

The electrochemical reaction is started by the movement of ions in an electric field. The identification of problems with electric field management resulting either from the simple design of electrode structures or from *in-situ* control of dendrite growth. The local areal current density will be significantly reduced by a well-designed anode structure or a 3D porous nanostructured anode, which will also result in less polarization and uniform deposition. The complex construction of the current collector will change the electric field’s uniform distribution, which will cause lateral growth rather of the vertical accumulation that has been observed in Li metal anodes. The nature of the electric field is also utilized for controlling advance charge/discharge protocols for removing dendrites with the intricate design (Yang et al., 2020).

**FIGURE 6:**
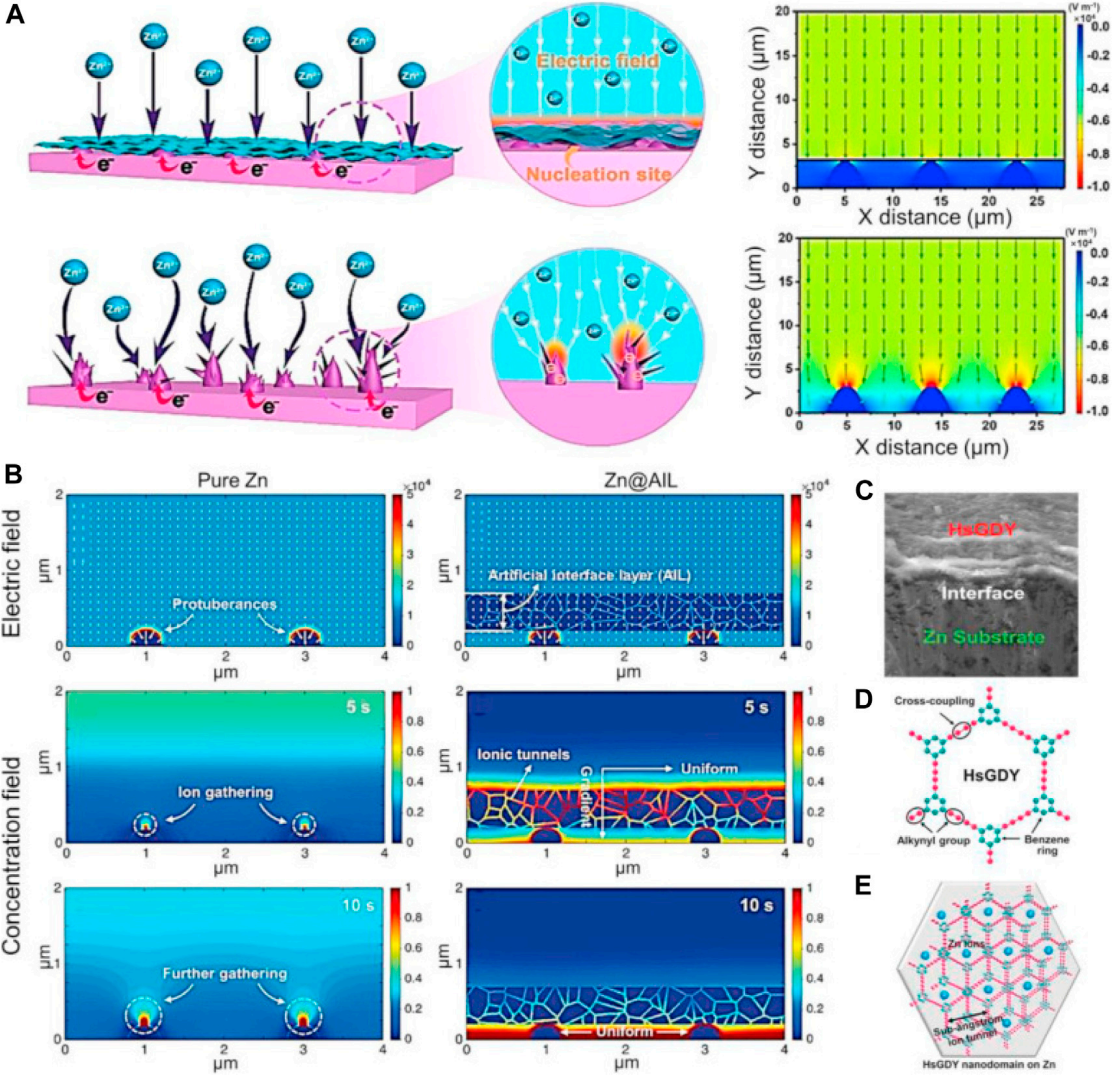
**(A)** Schematic representation of the behavior of zinc plating on pure zinc and zinc coated with MXene, together with the associated models of electric field distributions. Diagrams of the HsGDY and its sub-angstrom ion tunnel are shown in **(B)** dual-field simulations of pure Zn and Zn@HsGDY, **(C)** Zn@HsGDY cross-section, and **(D,E)**. CC-BY Creative Commons Attribution 4.0 International license was used to permit this reproduction (Chu et al., 2022). Copyright (2022), Elsevier.

Another efficient strategy for facilitating highly uniform Zn^2-^ distribution is to encourage uniform electric field distribution. Thus, conductive carbon materials have frequently been added to Zn anodes to increase the electro-active surface area, further homogenize the electric field distribution, and reduce Zn dendritic growth. These materials include porous carbon film, carbon nanotube (CNT) scaffolds, reduced graphene oxide (rGO), carbon black, and graphite. In addition, layer-by-layer self-assembled MXene layers were created on Zn anodes in order to create ultrathin and uniform MXene layers and efficiently homogenize the electric field distribution as shown in Figures 6A,B). In order to shift the Zn^2−^concentration field and completely eradicate Zn dendrites, Zhi et al. demonstrated that hydrogen-substituted graphene (HsGDY) may be connected with a Zn electrode (Figures 6C–E).

#### 4.1.1 3D current collector/electrodes

Batteries, which focus on a 2D confusion and nucleation, typically use a planar current collector. In comparison to a planar structure, all 3D architectures should pay special attention to their bigger electroactive regions (Cao et al., 2020).

#### 4.1.2 Charge/discharge protocols

Charge/discharge techniques, which are significantly more measurable and cost-effective, introduce specific *in situ* charge/discharge processes to modify dendrite formation. A recent paper described an electro-healing technique that combined cycling at high current density with a low current density stripping/plating process. Without giving the batteries any extra care, those techniques have a significant impact on applications and will pave the way for battery maintenance. To support the proposal, it is important to investigate the anode’s future long-term cycling impact and its viability with the available infrastructure (Huy, Hieu, and Hur 2021).

### 4.2 Ion flux regulation

The ion flux in the electrolyte can be influenced by numerous complex parameters, in contrast to the simple electric field control (Yu et al., 2020). The electrolyte, interfacial layer, and separator, along with other mass transfer processes occurring close to the interface while deposition takes place, have a significant impact on the ion movement caused by the electric field. We can get a consistent ion migration behavior and reduced polarization at the dendrite suppression by changing the electrolyte’s constituents and concentration (Wang et al., 2022). Aqueous and nonaqueous electrolytes can be used to classify the electrolyte (including ion liquid electrolytes and organic liquid electrolytes) (Zhang et al., 2022). According to PH, the majority of the electrolytes in RZBs are aqueous-based (including hydrogel and water-in-salt) and are classified as alkaline, neutral, or mildly acid. We concentrate on the aqueous-based electrolytes for RZB in this section. Meanwhile, interfacial modification can work as an ion filter for uniform movement and deposition as well as directly shield the anode from parasitic reaction. For a homogeneous plating, the functionalized separator will also promote uniform ion dispersion (Pan et al., 2018).

#### 4.2.1 Salts and additives

The primary salts and additions are included in the electrolyte components. Two common salts, ZnSO_4_ and Zn (CF_3_SO_3_)_2_, have variable electrochemical properties depending on their stability, conductivity, and compatibility. To be more precise, Zn(CF_3_SO_3_)_2_ makes a difference in the potential hysteresis between plating and stripping. This is due to the large CF_3_SO_3_ anions, which will reduce the solvated sheath surrounding Zn^2+^ and aid Zn^2+^ migration. Even though Zn(CF_3_SO_3_)_2_ has a stronger electrochemical performance, ZnSO_4_ is the salt that offers the greatest potential for cost savings and environmental benefits (Zhu et al., 2021).

#### 4.2.2 Water in salts electrolyte

Better electrolyte concentrations typically result in higher conductivity and lessened polarization in addition to the components. Another benefit of concentration is the destruction of the Zn^2+^solvation-sheath structure, which is linked to high Zn reversibility and is known as high concentration Zn ion electrolyte (HCZE) or water in salts (WIS) (Pan et al., 2016).

#### 4.2.3 The hydrogel electrolyte

The most promising electrolyte for wearable technology is called a hydrogel electrolyte, which is a network of polymer chains with embedded electrolyte. It can provide improved mechanical strength, ion confinement, and ion dispersion by adding functional groups to the polymer chain. The functionalization can also cause an even deposition at the contact and control ion flux. Hydrogel electrolytes must carefully balance their ion conductivity and mechanical strength in order to withstand large current densities. On the other hand, water retention and stability will also be taken into account when commercialization is being considered (Zhang et al., 2016). Ling et al. (2021), reported Self-healable hydrogel electrolyte for dendrite-free and self-healable zinc-based aqueous batteries, which delivered a high capacity of 304 mAh·g^−1^ at 0.5 A·g^−1^ and good cycling stability with a capacity retention of 83.1% (vs. 62.5% with polyacrylamide) after 1,500 charge/discharge cycles at 5.0 A·g^−1^.

#### 4.2.4 Surface coating

**FIGURE 7:**
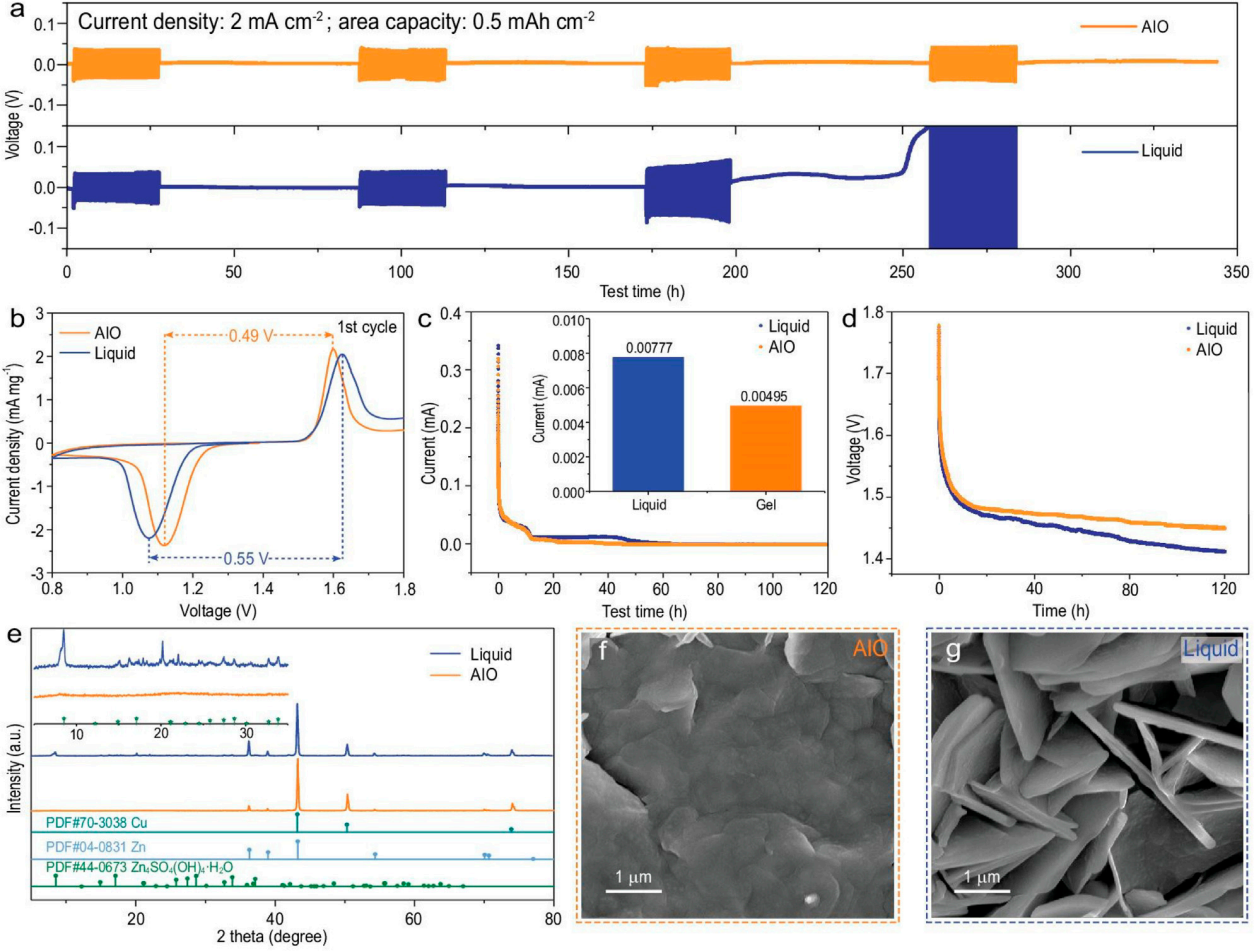
**(A)** Shelving-recovery performance of Cu foam@Zn/Cu foam@Zn symmetric cell (AIO electrode/Cu foam@Zn in AIO system) under 2 mA·cm^−2^. **(B)** First cyclic voltammetry curve, **(C)** float charge current, **(D)** open circuit potential decays of AIO electrode/α-MnO_2_ and Cu foam@Zn/α-MnO_2_ full cell. **(E)** XRD patterns of the anodes in different full cell systems after 100 cycles at 500 mA·g^−1^, and the corresponding SEM images of **(F)** AIO electrode and **(G)** Cu foam@Zn in 2 M ZnSO_4_ + 0.1 M MnSO_4_. Reproduced with permission (Li et al., 2022). Copyright (2022), Oxford University Press.

The most effective defense against dendritic problems for metal anodes is surface coating, which is often created through doctor blading, spin coating, and atomic layered deposition (Wang et al., 2018). There are two types of coatings: organic and inorganic. The formal one often serves as a barrier against adverse effects and offers a consistent ion route. Canpeng Li et al., developed a “all-in-one” (AIO) strategy by combining structural design, interface modification, and electrolyte optimization, inheriting the benefits of the 3D zinc anode and gel electrolyte with nearly no hydrogen evolution (Figure 7).

#### 4.2.5 Separator modification

A separator is a crucial component in the prevention of short circuits. The ion flux will also be redistributed as a result. Currently, there are two main types of separators: nonwoven paper and glass fiber (Jia et al., 2020). Due to its great thickness and lack of economics, glass fiber has a high degree of resilience and chemical inducing to prevent dendrites. The commercialization of RZBs is economically aided by the nonwoven paper, however this paper’s worst property is that it is easily pierceable. A good mechanical property, low operating expenses, and improved ion flux guiding are desirable characteristics in a separator that inhibits dendrite formation (Zhang et al., 2017).

### 4.3 Mechanical shield

The mechanical elements will surely have an impact on the dendritic formation and short circuit process. There has not been any systematic research or simulation too far for RZBs to estimate this threshold (Li et al., 2020a). The crucial function of physical shielding can be seen in the apparent morphological difference between the deposition in the electric tank and a cell with a separator. The anhydrous property and increased mechanical strength should stabilize the Zn anodes. The problems to be solved in further research will be the low ionic conductivity and poor interfacial contact that are accompanied by high polarization. The operation of mechanical shielding is frequently coordinated with other surface coating, gel and solid-state electrolyte, and enhanced separator methods. The balance between mechanical property and electrochemical property, such as ion conductivity, and other factors, should be taken into consideration while creating robust physical shielding (Wang et al., 2019).

### 4.4 Crystallographic orientation manipulation

Zn is a crystalline substance, and the preferred crystallographic orientations can be changed throughout the electrodeposition process to control the surface texture. A dense and parallel orientation is what a properly produced Zn anode should have, and this can be accomplished by adjusting the substrate used and the electrodeposition electrolyte additive. While the substrate is modified *via* epitaxial electrodeposition, the electrolyte additive modifies the crystallographic by chemical contact (Zhang et al., 2020). The following Zn deposition tends to be oriented similarly during the battery cycling process as a result of the lattice match, which contributes to better electrochemical performance. The initial Zn anode can be controlled using straightforward techniques, making it feasible to produce stable Zn anodes in large quantities. However, the efficiency after prolonged cycling needs to be further assessed (Shin et al., 2020).

### 4.5 Engineering of substrate

#### 4.5.1 Engineered materials and substrate selection

For dendritic suppression, it is crucial to select the right substrate or modified materials. In order to achieve efficient electron transport, the substrate for zinc deposition must be insoluble in aqueous electrolyte. Currently, porous carbon materials, copper foam, nickel foam, and foil are the most often utilized substrates. On the other hand, a crucial aspect of substrates’ characteristics is their zinc nucleation overpotential (Yang et al.). Low nucleation overpotential will result in a smaller potential barrier to overcome, allowing for uniform zinc deposition and improved zinc plating/stripping reversibility. Cu foam has been proven in prior research to have a number of inherent advantages as a substrate, and various modification techniques can further optimize zinc nucleation overpotential. The excellent adsorption ability of zinc atoms is advantageous for the uniform deposition when choosing modified materials. Stronger interactions between zinc and surface particles with strong electron interactions and polarization can both prevent zinc from aggregating on the substrate surface and from flaking off once it has been deposited (Mainar, Blazquez, and Urdampilleta 2017).

#### 4.5.2 Substrate surface engineering

**FIGURE 8:**
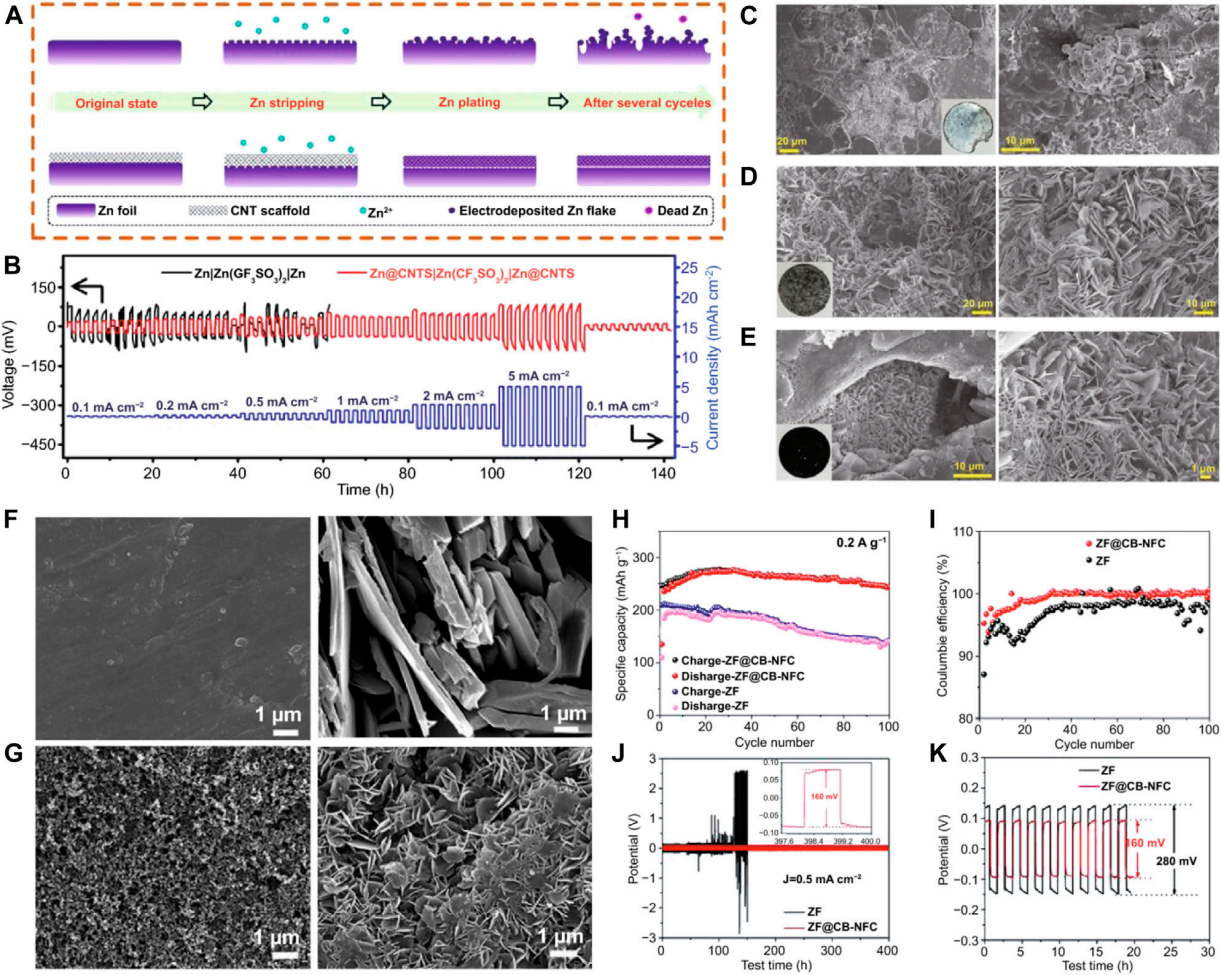
**(A)** Diagrams illustrating the stripping/plating behaviors of Zn anodes stabilized by CNT scaffolds and bare ZF anodes. **(B)** Rate performance over a 1-h period at varying current densities of 0.1–5 mA·cm^−2^. **(C)** Symmetrical Zn||Zn cells’ Zn electrodes as seen in a SEM picture. **(D)** SEM picture of symmetrical Zn@CNTs cells with Zn electrodes. **(E)** SEM picture of symmetrical Zn@CNT cells with CNT scaffolds (after cycling tests). SEM pictures of a ZF anode and a ZF@CB-NFC anode are shown in **(F,G)**, respectively. **(H)** Cycling accomplishments at 0.2 A·g^−1^. **(I)** The relationship between Coulombic efficiency and cycle number. **(J)** Galvanostatic charge/discharge (GCD) curves for Zn symmetrical cells with ZF and ZF@CB-NFC electrodes, 200 cycles at 0.5 mA·cm^−2^. **(K)** Zn symmetrical cells with ZF and ZF@CB-NFC electrodes: voltage profiles of the 1st–10th cycles at 0.5 mA cm^−2^. Creative Commons Attribution 4.0 International license was used to permit this reproduction (Li et al., 2022). Copyright (2021), Springer.

Metal affinity functional groups frequently control the surface of the optimal substrate, resulting in uniform locations for corresponding metal deposition (Qin et al., 2021). Zinc will be deposited uniformly as a result of the improved coatings’ capacity to optimize the electron transport channel, the electric field distribution, the wettability of the interface, and other factors (Cui, Han, and Hu 2021). Moreover, by controlling Zn^2+^ deposition, a surface coating is designed to provide uniform Zn nucleation and a flat Zn deposition layer, which significantly enhances the Zn anode’s interface stability and cycle lifetime (A. Wang et al., 2020). Previous reported results state that a variety of materials, including carbon-based materials, metal materials, inorganic non-metals, polymers, and composite materials, have been employed as interfacial layers to produce high-performance Zn anodes (Li et al., 2018). As a widely used one-dimensional material, CNTs are lightweight and have excellent chemical properties. Yang et al. (Dong et al., 2020) developed self-supporting, extremely flexible, and conductive CNT/paper scaffolds to stabilize Zn metal anodes because unaltered Zn forms dendrites that lead to unfavorable interactions at the Zn anode/electrolyte interface, ultimately resulting in the failure of RAZIBs (Figure 8A). The porous scaffold’s skeleton mechanically controlled where Zn^2+^ would deposit on the Zn electrode’s surface, while the conductive CNT network kept the electric field uniform (Zuo et al., 2021). A more stable charge/discharge behavior was also shown by the built Zn@CNT symmetrical cells (Figure 8B). After cycling, the modified ZF didn’t exhibit any notable modifications (Figures 8C,D). Zn anodes were modified by Chen et al. (A. Wang et al., 2020) with nanofibrillar cellulose adhesives and C black coatings. The dendrite formation and side reactions on the anode are minimized by altering zinc foil with a C black coating and a nanofibrillating cellulose (NFC) binder, resulting in excellent interfacial stability between the anode and electrolyte. After 100 cycles, the ZF’s surface was covered in many dendrites (Figure 8F). Before and after cycling, the anode made of ZF modified with a C black coating and NFC binder (ZF@CB-NFC) maintained a consistent surface (Figure 8G). The redesigned anode-based cell exhibited improved cycle stability and CE (Figures 8H,I). The redesigned Zn anode-based symmetrical cell demonstrated improved cycle performance and a lower polarization voltage (Figures 8J,K). After circulation, metrical cells had the same appearance (Figure 8E).

#### 4.5.3 3D porous substrate

High current density, as was already established, is detrimental to zinc’s uniform deposition. The substrate’s specific surface area can be effectively increased by the 3D porous structure, which lowers the local current density (Zhang et al., 2021). As a result, the anode and electrolyte have a larger surface area in contact with each other and the current density is distributed uniformly. Additionally, it is crucial for controlling ion transfer, preventing dendritic development, and preserving the battery’s dimensional stability. As a result, there is less chance that dendrites will form (Bayaguud, Fu, and Zhu 2022). The inhibition of zinc dendrite and the homogeneous distribution of zinc are both made possible by the aforementioned benefits. Therefore, structural modification is a useful strategy for reducing dendritic development (Higashi et al., 2016). These recently discovered methods for preventing zinc dendrites are demonstrated, together with the accompanying electrochemical performance (Table 1).

**TABLE 1:** An overview of recently reported dendrites free engineering strategies of zinc-based anode materials.

Engineering strategies of anode materials	Limit capacity [mAh cm^−2^]	Current density [mA cm^−2^]	Lifespan [h]	Voltage hysteresis [mV]	Ref
Nano-TiO_2_ coating on zinc anode	-	0.4	500	50	Li et al. (2021a)
Al_2_O_3_-coated zinc plate	1	1	500	36.5	He et al. (2020)
HfO_2_-coated zinc anode	1	1	500	63	Li et al. (2021b)
CaCO_3_-coated zinc foil	0.05	0.25	836	80	(Kang et al., 2018)
ZrO_2_-coated zinc foil	1	5	2,100	32	Zhao et al. (2020)
3D zinc anode@carbon fibers	1	1	350	30	(Dong et al., 2018)
3D flexible carbon nanotubes	2	2	200	27	Shi et al. (2020)
3D porous copper skeleton	0.5	0.5	40	350	(Kang et al., 2019)
Zn_88_Al_12_ alloys	0.5	0.5	2000	≈20	Wang. (2020)

## 5 Summary and perspective

In conclusion, it is critical to recognize that the RZBs are the most viable candidate to construct a low-cost, secure system with adequate capacity, particularly for wearable devices. The main issues with the Zn anode, including as dendritic growth, hydrogen evolution, and passivation, are succinctly summarized in this paper. The most serious issue that might cause battery failure and safety problems is the dendrite problem. As a result of the previous discussion, there are four categories for dendritic suppression strategies: Electric field control involves designing 3D electrodes for low local current density and removing dendrite tips according to predetermined protocols; ion flux regulation involves introducing electrostatic interaction, adsorption, and changing the solvent shear structure; mechanical shield involves creating a strong interface by the use of designed protocols for the initial Zn anode. While each of those approaches can be used independently to improve performance, many approaches can be used simultaneously. An illustration of this is how a metal oxide surface coating will result in a consistent ion flux distribution and act as a physical barrier to stop dendrite formation. The increased electrochemical performance and extended anode lifespan are wonderful developments. Theoretical research and fundamental understanding, however, are still in the early stages and require more study. As a result, difficulties persist and a number of issues need to be taken into account.

Moreover, the problem of zinc dendrites to the ground is typically not resolved by using additions for electrolytes and electrodes, especially in alkaline mediums. In this situation, it is important to use efficient techniques to reduce or even eliminate zinc dendrites’ negative impacts, specifically by eliminating battery shorts as much as possible. Separators need to have a high level of mechanical stability to prevent impalement, as well as a high level of chemical stability to prevent degradation.

These elements will need to be taken into account in the future effort, and the following guidelines are strongly advised:

• A combination of theoretical direction and real-world requirements should be used to design the test conditions and standards. There is enough information to conclude that the current density is influenced by application situations and manufacturing capability, which both determine the mass loading range. The test environment should make an effort to mimic commercial requirements and machine capabilities.
• Future optimization should address several issues simultaneously using a variety of methodologies. For mass manufacturing to be feasible under the conditions of acceptable cost, the strategies should be properly integrated. For instance, the long-term stability is ensured by using affordable electrolyte additives to moderate the ion flux and a thin surface layer for physical shielding and corrosion prevention. The additives are placed in a hydrogel electrolyte with great mechanical strength and homogenous ion channels for dendrite suppression.
• Cutting-edge technologies, such as *in-situ* XRD for detecting the prevailing crystallographic orientation and *in-situ* optical/X-ray microscopy for morphological variation, enable the fundamental theory to monitor the interfacial process of the Zn anode *in situ*.
• Theoretical investigation showed that, different methods and strategies are used to stop uneven deposition. The creation of a stable Zn anode will be substantially accelerated by having a holistic awareness of how to recognize the crucial elements under various conditions.
• The most widely used techniques to completely and permanently inhibit the formation of zinc dendrites up until now have included designing a 3D porous structure with high zinc-based electrode surface areas, using acidic or neutral mediums, ionic liquids along with other novel mediums as electrolytes, and a combination of these techniques. The successful development of a dendrite-free zinc deposition procedure when using these techniques in zinc-based batteries would improve their performance and advance their business model.
